# Loss of function of Noggin inhibits glial scar formation and motor function recovery after spinal cord injury

**DOI:** 10.3389/fncir.2026.1821905

**Published:** 2026-05-28

**Authors:** Yibo Han, Yuki Wakayama, Shuo Li, Dong Liang, Juntan Li, Yashuang Ping, Hideyuki Arima, Kohji Sato, Yukihiro Matsuyama, Satoru Yamagishi

**Affiliations:** 1 Department of Organ and Tissue Anatomy, Hamamatsu University School of Medicine, Hamamatsu, Japan; 2 Department of Orthopedic Surgery, Hamamatsu University School of Medicine, Hamamatsu, Japan; 3 Department of Orthopedics, Dalian Municipal Central Hospital, Dalian, China; 4 Department of Optical Neuroanatomy, Institute of Photonics Medicine, Hamamatsu University School of Medicine, Hamamatsu, Japan; 5 Anorectal Disease Hospital, The Third Affiliated Hospital of Henan University of Chinese Medicine, Zhengzhou, Henan, China; 6 Department of Cellular and Molecular Anatomy, Hamamatsu University School of Medicine, Hamamatsu, Japan

**Keywords:** antibody neutralization, astrocyte, BMS score, glial scar, Noggin, spinal cord injury

## Abstract

**Introduction:**

Noggin is a glycosylated protein that acts as an antagonist of bone morphogenetic proteins and has been implicated in astrogliosis and functional recovery after spinal cord injury (SCI). Although Noggin is known to regulate astrocytic responses, its role in reactive astrogliosis and glial scar formation after SCI remains unclear. Under normal conditions, Noggin is expressed in astrocytes in the white matter of the mouse spinal cord. In this study, we investigated the role of Noggin in reactive astrogliosis and glial scar formation after SCI.

**Methods:**

We first examined the expression pattern of Noggin in the mouse spinal cord after SCI. To assess the functional role of Noggin, we used *Nestin-Cre; Noggin^flox/flox^* conditional knockout mice, in which Noggin is conditionally deleted from astrocytes and neurons in the spinal cord. In addition, neutralizing anti-Noggin antibodies were continuously administered to the lesion site using micro-osmotic pumps. GFAP expression and motor function after SCI were then evaluated.

**Results:**

As a result, we found that Noggin was upregulated in reactive astrocytes after SCI, suggesting its involvement in the injury response. Conditional deletion of Noggin markedly suppressed glial fibrillary acidic protein (GFAP) expression after SCI. Consistently, local administration of neutralizing anti-Noggin antibodies also reduced GFAP expression around the lesion site. However, anti-Noggin antibody treatment deteriorated motor function after SCI.

**Discussion:**

These findings suggest that Noggin is an important regulator of reactive astrogliosis and contributes to glial scar formation after SCI. Although suppression of Noggin reduced GFAP expression and attenuated glial scar formation, it did not improve functional recovery. Rather, motor function was worsened, indicating that reduced glial scar formation may not necessarily be beneficial after SCI. Noggin-mediated astrogliosis may therefore play a protective role in the injured spinal cord.

## 1 Introduction

SCI triggers immediate mechanical disruption followed by a prolonged secondary injury cascade that includes vascular compromise, inflammation, excitotoxicity, and progressive tissue remodeling. These processes collectively shape the lesion core and the surrounding peri-lesional border, which strongly influence neurological outcome. A hallmark of SCI pathology is the formation of a glial scar—a dynamic cellular and extracellular interface composed primarily of reactive astrocytes, activated microglia/macrophages, and a remodeled extracellular matrix (ECM) (Pajer et al., 2020; Tian et al., 2023). The glial scar has long been considered a major impediment to axon regeneration because it forms a physical barrier and is enriched in inhibitory ECM molecules such as chondroitin sulfate proteoglycans (CSPGs) (Silver and Miller, 2004). At the same time, it is increasingly appreciated that scar-forming astrocytes also provide essential protective functions, including restricting inflammatory spread, stabilizing tissue, and limiting lesion expansion, particularly in the acute and subacute phases (Sofroniew, 2018; Liddelow et al., 2024). Thus, delineating the molecular mechanisms that govern reactive astrocyte states and their interactions with immune cells is critical for rationally targeting the injury environment to promote repair while preserving tissue-protective functions (Pajer et al., 2020; Tian et al., 2023).

Reactive astrogliosis is characterized by astrocyte hypertrophy, altered gene expression, and induction of intermediate filament proteins such as GFAP (Sofroniew, 2018). These changes culminate in the assembly of an astroglial border that demarcates the lesion margin and contributes to scar maturation over time (Bradbury and Burnside, 2019). In parallel, microglia and infiltrating macrophages accumulate within and around the lesion, where they participate in debris clearance but can also amplify tissue damage through sustained inflammatory signaling (Xu et al., 2022). The balance between astroglial border formation and myeloid cell accumulation is a key determinant of lesion containment, ECM remodeling, and the permissiveness of the peri-lesional region for axon preservation or sprouting (Bradbury and Burnside, 2019; Tian et al., 2023). However, the signaling pathways that coordinate these astrocyte–myeloid dynamics in the injured spinal cord remain incompletely understood.

Bone morphogenetic protein (BMP) signaling is a candidate pathway with well-established roles in neural development and injury responses (Re’em-Kalma et al., 1995; Al-Sammarraie et al., 2023). BMP ligands engage type I and type II serine/threonine kinase receptors to activate downstream SMAD1/5/8 phosphorylation, driving transcriptional programs that influence cell fate and glial differentiation (Re’em-Kalma et al., 1995; Sawant et al., 2012). After central nervous system injury, BMP pathway components and BMP-responsive signaling activity can increase, and BMP signaling has been implicated in promoting astroglial reactivity and ECM changes that are relevant to scar formation (Al-Sammarraie et al., 2023). Notably, genetic manipulation of BMP type I receptors has demonstrated that individual BMP receptor subunits exert distinct effects on gliosis and immune cell recruitment in SCI models, underscoring the complexity and context-dependent nature of BMP pathway regulation *in vivo* (Duan et al., 2015). These findings motivate a closer examination of endogenous mechanisms that regulate BMP signaling specifically within the post-injury microenvironment.

Noggin is a secreted endogenous antagonist that binds BMP ligands and prevents receptor activation, thereby acting as an important negative regulator of BMP signaling (Smith and Harland, 1992; McMahon et al., 1998). In developmental contexts, Noggin plays a central role in shaping neural tissue patterning through BMP inhibition, and experimental modulation of BMP–Noggin balance can influence glial lineage outcomes (McMahon et al., 1998; Lim et al., 2000). In the context of SCI BMP signaling can bias progenitor and glial responses toward astrocytic fates, while BMP antagonism can shift injury-associated cellular programs and potentially affect repair-related outcomes (Matsuura et al., 2008; Xiao et al., 2010; Al-Sammarraie et al., 2023). Although improved outcomes after SCI have been reported following Noggin administration (Matsuura et al., 2008), the endogenous functions of Noggin in SCI have not been directly tested using knockout or suppression strategies. Furthermore, previous studies have reported contradictory effects of Noggin administration on glial scar formation, and each BMP receptor have distinct functions (Sahni et al., 2010; Xiao et al., 2010; Al-Sammarraie et al., 2023). Consequently, the role of Noggin in glial scar formation havs not been clearly understood.

In this study, we investigated the relationship between Noggin signaling and lesion development and glial scar formation after SCI, as well as the requirement for Noggin activity in typical astroglial and inflammatory responses. Using *Nestin-Cre; Noggin^flox/flox^* conditional knockout (cKO) mice, we discovered that eliminating Noggin from astrocytes and neurons in the spinal cord strongly suppresses GFAP expression after SCI. Furthermore, the administration of neutralizing anti-Noggin antibodies to the lesion site via micro osmotic pumps inhibited GFAP expression and deteriorated motor function following SCI. These findings suggest that Noggin is a key regulator of astrogliosis and plays an important role in glial scar formation. However, decreased glial scar formation may not improve motor function after SCI.

## 2 Materials and methods

### 2.1 Animals

*Nog-GFP* BAC transgenic mice were obtained from GENSAT and maintained on a CB17 background. Adult (>8 weeks old) *Nog-GFP* mice were used. Noggin-floxed mice were obtained from the Jackson Lab (Strain #016117) (Stafford et al., 2011). C57BL/6-Tg (Nestin-Cre) 1Kag mice were obtained from CARD mouse Bank, Kumamoto University. To generate cKO mice, *Noggin^flox/flox^* mice were crossed with Nestin-Cre (Isaka et al., 1999). Primer pairs for genotyping were as follows: Nog-GFP, 5′-GGG GGG AAT TGC GAC CAA-3′ and 5′-TAG CGG CTG AAG CAC TGC A-3′; Noggin^flox^, 5′-CCA CAA TAT CCA GCC CTT GT-3′ and 5′- AAG AGG CCC ATG TGA GTG TC-3′; and Cre, 5′-GCCTGCATTACCGGTCGATGCAACGA-3′ and 5′-GTGGCAGATGGCGCGGCAACACCATT-3′. All animal experiments were approved by the Animal Research Committee of Hamamatsu University School of Medicine and were performed in accordance with the in-house guidelines for the care and use of laboratory animals of the university.

### 2.2 SCI

Female mice were deeply anesthetized with medetomidine hydrochloride (7.5 μg/10 g), midazolam (40 μg/10 g), and butorphanol tartrate (50 μg/10 g), and laminectomy was performed at the ninth and tenth thoracic (T9–T10) levels. Subsequently, a contusion SCI was induced at T9 using the Infinite Horizons impactor (Muromachi, Tokyo, Japan) with an impact force of 60 kdyn, as described previously (Arima et al., 2014). The skin was then closed with wound clips. Postoperatively, the antagonist atipamezole hydrochloride (7.5 μg/10 g) was injected intraperitoneally in an equal amount as the anesthetic. After ascertaining awakening, mice were housed in cages with drinking water spiked with antibiotics (0.5 mL of Bactramin in 200 mL of water) for 7 days to prevent infection. Animals were sacrificed on days 3, 7, and 14 post-SCI for immunostaining.

### 2.3 Behavioral assessment

Motor recovery in the hind limbs of mice was assessed using the Basso mouse scale (BMS) open-field locomotor test before SCI and on days 1, 3, 7, 10, 14, 21, and 28 post-SCI (Basso et al., 2006). The BMS score was assessed independently by two operators who were unaware of each group, and the final scores of both hind limbs were averaged.

### 2.4 Tissue preparation

Mice were deeply anesthetized and fixed by cardiac perfusion with sodium phosphate-buffered saline (PBS; pH 7.4) for 1 min followed by 4% paraformaldehyde in PB (pH 7.4) for 5 min. Spinal cords were dissected and placed in cold 4% paraformaldehyde overnight and then in a sequence of 15% and 30% sucrose/PBS for cryoprotection. The samples were embedded in optimal cutting temperature (OCT) compound at −80 °C or in paraffin. They were then cut into sagittal sections for hematoxylin and eosin (HE) staining and immunohistochemistry.

### 2.5 Immunostaining

Sections were dried, soaked in 4% paraformaldehyde for 10 min, washed with PBS, and permeabilized in 0.3% Triton X-100/PBS for 5 min. The sections were incubated in blocking solution containing 3% bovine serum albumin in 0.1% Triton X-100/PBS for 1 h at room temperature and then incubated with primary antibodies in blocking solution overnight at 4 °C. For 3,3′-diaminobenzidine (DAB) staining, the sections were incubated with the anti-rabbit Envision+ System Horseradish Peroxidase (DAKO, Santa Clara, USA) for 30 min at room temperature followed by development using the ImmPACT DAB Substrate Kit (Vector, Newark, USA). The sections were imaged using a transmitted light microscope (Eclipse E600; Nikon, Tokyo, Japan). For fluorescent immunostaining, sections were incubated with primary antibodies followed by Alexa-Fluor-conjugated secondary antibodies for 30 min at room temperature. Then they were processed for nuclear staining with DAPI. Images were acquired using a confocal microscope (TCS SP8; Leica, Wetzlar, Germany). The primary polyclonal antibodies used were rabbit anti-GFP (1:1,000; No. 598; MBL), rabbit anti-Noggin (1:350; ab16054, Abcam), goat anti-Noggin (1:500; AF719, R&D Systems), goat anti-Iba1 (1:1,000; ab107159, Abcam), rabbit anti-Ki67 (1:500; ab16667, Abcam), rabbit anti-phospho-SMAD 1/5/8 (1:500, #9511, Cell Signaling Technology), rabbit anti-NG2 (1:500; ab259324, Abcam) and goat anti-CD206 (1:500; AF2535, R&D Systems). Secondary donkey anti-rabbit and anti-goat antibodies (conjugated to Alexa Fluor-488, -594, and -647; Thermo Fisher Scientific) were used at a dilution of 1:500. Mouse monoclonal anti-GFAP conjugated to Cy3 (1:1,000; C9205, Sigma) was also used to visualize astrocytes.

### 2.6 3D imaging

We followed CUBIC protocols (Susaki et al., 2015). In brief, we treated spinal cord with CUBIC-L and stained with GFP-Booster antibody (1:200, gba488; Chromotek) and anti-GFAP antibody conjugated with Cy3 (1:200; C9205, Sigma). Samples were processed for RI matching with CUBIC-R+. Images were acquired using light-sheet microscope (Lightsheet7; ZEISS).

### 2.7 Anti-noggin antibody treatment

We used rabbit anti-Noggin antibody (ab16054, Abcam) to neutralize Noggin. The day before SCI, the antibody (23.3 μg/kg/day over 2 weeks) or control rabbit IgG (23.3 μg/kg/day over 2 weeks; Sigma-Aldrich) was administered with an osmotic pump (200 μL solution, 0.5 μL/h, 14 day delivery; Alzet pump model 2002, Durect Co., Cupertino, USA) (Nakanishi et al., 2019). The pumps were immersed in 37 °C saline and incubated overnight. After SCI, the pumps were placed under the back skin of the mice, and polyethylene tubes connected to the pumps were placed above the lesion site. The tubing was secured with sutures to the muscle caudal to the laminectomy. The skin was then closed with wound clips.

### 2.8 Quantification and statistical analysis

We took squares of equal area on high-magnification images (×63) along the midline of the spinal cord (gray matter area) at uniform distances. We quantified the images using ImageJ, and calculated the positive area to determine positive coverage, with all values expressed as mean ± SE. Three sections per mouse were analyzed (Wanner et al., 2013; Pomeshchik et al., 2015). The statistical significance of differences between groups was tested using the unpaired Student’s *t*-test, and *p* < 0.05 was considered significant. Histograms and trend plots were made and statistical analyses were performed using Excel.

## 3 Results

### 3.1 Upregulation of noggin expression after spinal cord injury

**Figure 1:**
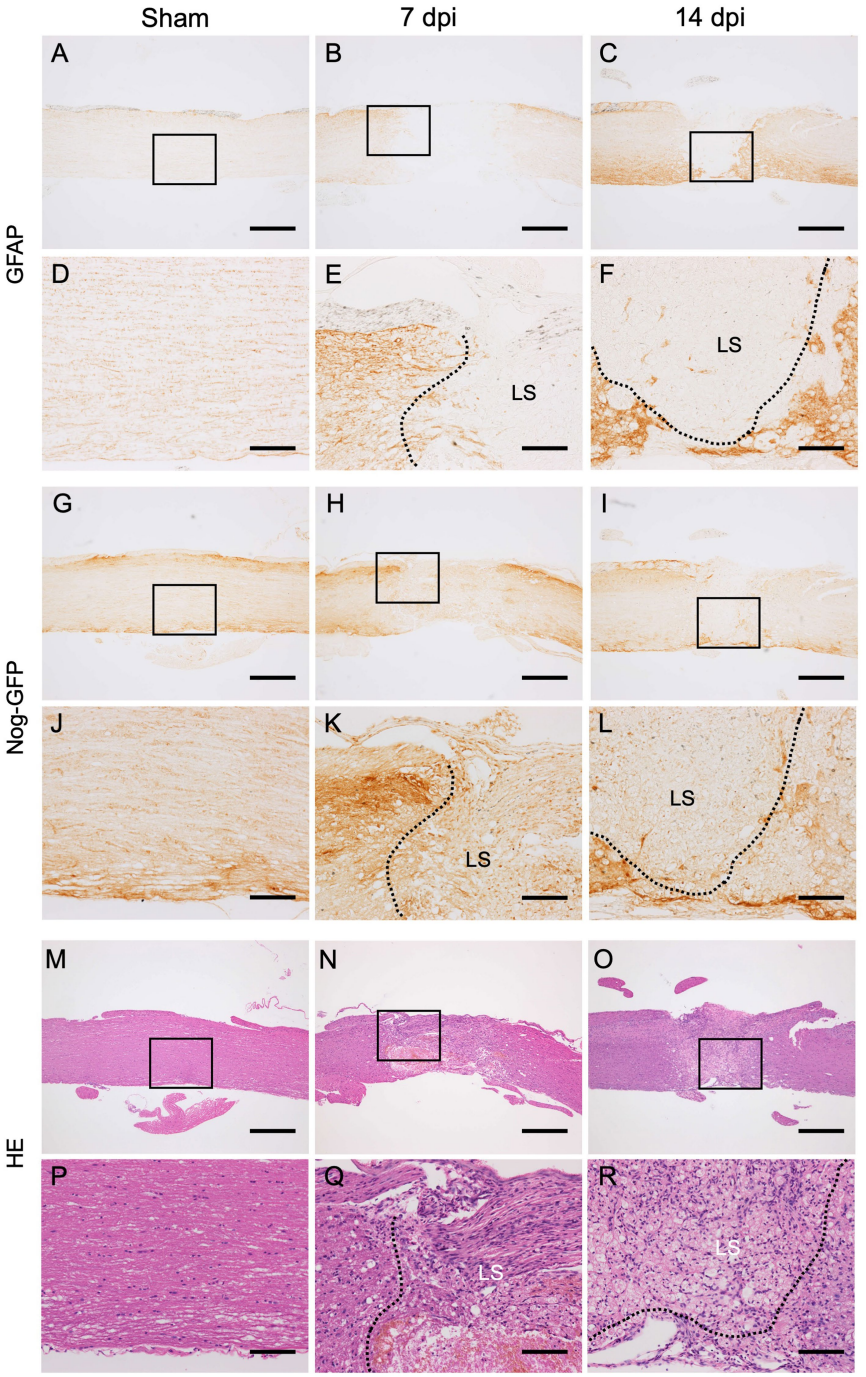
Upregulation of Noggin in gray matter after spinal cord injury. **(A–F)** Representative images of anti-GFAP antibody staining in the spinal cord of Nog-GFP transgenic mice in the sham group **(A,D)**, at 7 **(B,E)** and 14 days **(C,F)** after injury. **(G–L)** Representative images of anti-GFP antibody staining in the spinal cord of Nog-GFP transgenic mice in the sham group **(G,J)**, at 7 **(H,K)** and 14 days **(I,L)** after injury. **(M–R)** Representative images of HE staining in the spinal cord of Nog-GFP transgenic mice in the sham group **(M,P)**, at 7 **(N,Q)** and 14 days **(O,R)**. LS, lesion site. Dotted lines mark the injury boundaries. Boxes mark the regions of interest for high-magnification display. Scale bars: 500 μm **(A–C,G–I,M–O)** and 100 μm **(D–F,J–L,P–R)**.

To characterize the temporal changes that occur after SCI, we performed HE staining and immunohistochemistry for GFAP, as well as assessing Noggin expression using Noggin promoter–GFP reporter mice, which were detected by anti-GFP immunostaining (Figure 1). In animals that underwent a sham operation, the spinal cord architecture was preserved with no apparent tissue loss evident in HE staining (Figures 1M,P). Only low baseline GFAP immunoreactivity was observed. Noggin was expressed in the meninges and the fibrillar structure of the white matter of the spinal cord (Figures 1A,D,G,J). Weak expression of Noggin-positive fibrils was also observed in gray matter. GFP–positive GFAP-expressing astrocytes were enriched in the white matter compared with the gray matter (Supplementary Figure 1). To further validate the spatial distribution and cellular localization of Noggin-expressing astrocytes in the intact spinal cord, we performed three-dimensional immunofluorescence imaging of cleared uninjured spinal cord tissue using the CUBIC protocol. Consistent with the two-dimensional analyses, Nog-GFP and GFAP signals were predominantly distributed within the white matter and exhibited extensive co-localization in three dimensions (Supplementary Video 1). Consistent with previous reports, HE staining at 7 days post injury (dpi) revealed significant tissue architecture disruption accompanied by hemorrhagic/necrotic changes and pronounced vacuolation in the lesion area (Fleming et al., 2006) (Figures 1N,Q). By 14 dpi, the lesion had progressed to tissue rarefaction and cavitation, indicating ongoing secondary degeneration and remodeling (Figures 1O,R). In parallel with these histopathological changes, GFAP immunoreactivity increased in the tissue surrounding the lesion at 7 dpi, becoming more prominent by 14 dpi and forming a dense border of GFAP-positive cells around the lesion area (Figures 1B,C,E,F). This is consistent with a maturing astroglial response. Notably, anti-GFP staining in Nog-GFP mice revealed a weak signal in the control group, whereas a significant increase in the GFP signal was detected within and around the lesion at 7 dpi. This elevated signal persisted at 14 dpi and appeared enriched along the gliosis region (Figures 1H,I,K,L). Together, these findings suggest that SCI triggers time-dependent pathological progression, accompanied by reactive astrogliosis and sustained Noggin upregulation during the subacute phase, which is similar to the observation after dorsal rhizotomy (Hampton et al., 2007b).

### 3.2 Noggin is predominantly expressed in GFAP-positive reactive astrocytes at the lesion border

**Figure 2:**
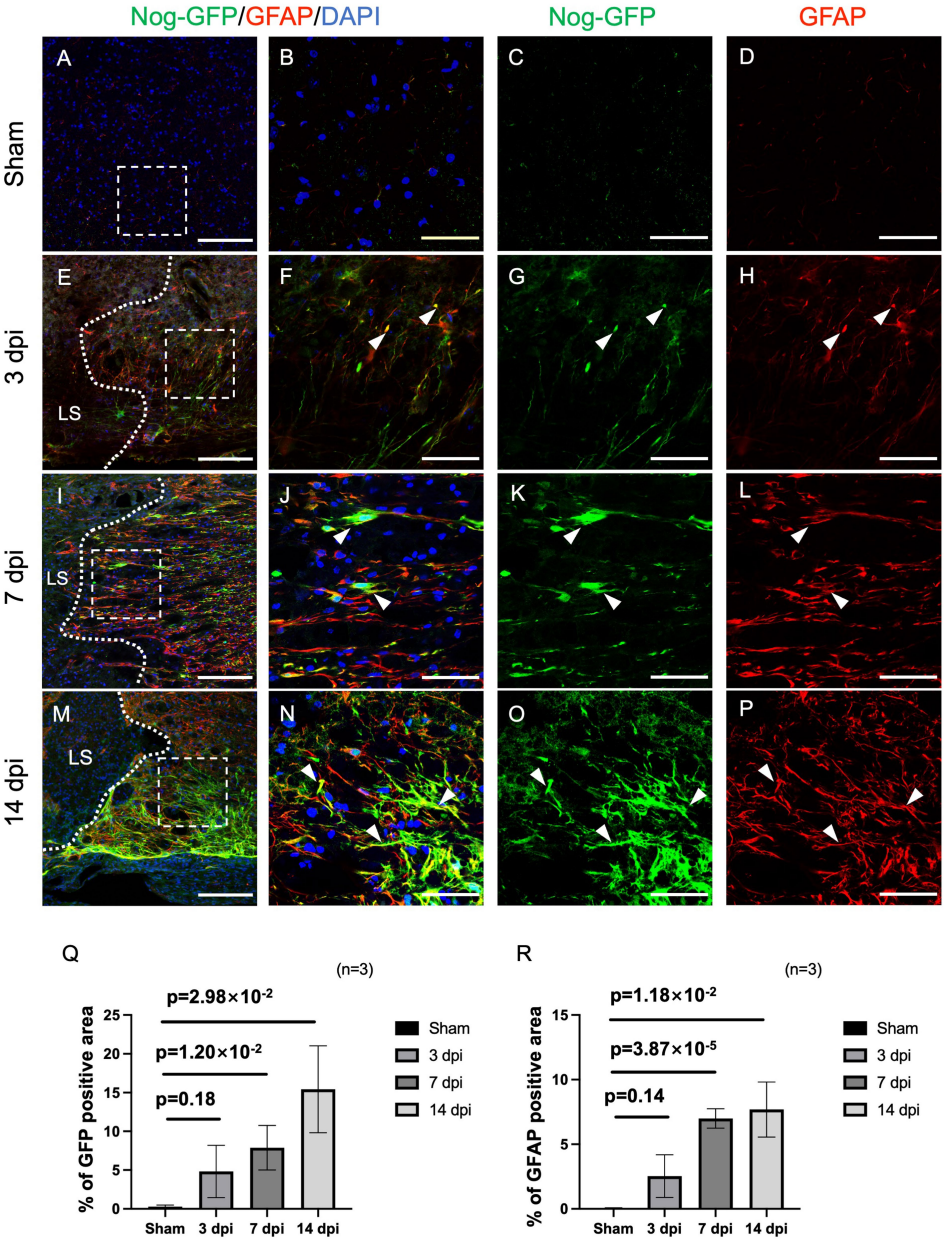
Astrocytic expression of Noggin after spinal cord injury. **(A–P)** Representative immunohistochemistry images showing GFP (green), GFAP (red), and DAPI (blue) in the spinal cord of Nog-GFP transgenic mice in the sham group **(A–D)** and after 3 **(E–H)**, 7 **(I–L)**, and 14 **(M–P)** days after injury. Most of the GFP positive cells after spinal cord injury were GFAP-positive (arrowheads in **F–H**, **J–L**, and **N–P**), whereas GFP immunoreactivity in gray matter after sham was very weak **(B–D)**. **(Q,R)** Quantification of the percentage of GFP- and GFAP-labeled regions. The mean of three sections was used for quantification (3 mice per group). Data are presented as mean ± SE. LS, lesion site. Dotted lines mark the injury boundaries. Dotted boxes mark the regions of interest for high-magnification display. Scale bars: 200 μm **(A,E,I,M)** and 50 m **(B–D,F–H,J–L,N–P)**.

To define the cellular localization of Noggin expression after SCI, we performed double immunofluorescence for GFP and GFAP across time points (sham, 3, 7, and 14 dpi). In the uninjured spinal cord, astrocytes are mainly located in white matter (Guttenplan and Liddelow, 2019), and a small number of astrocytes were present in gray matter with weak expression of Noggin (Figures 2A–D). After SCI, astrocytes undergo a transformation from a quiescent state to reactive astrocytes, which migrate toward the lesion site and form a reticular structure at the glial scar (Bradbury and Burnside, 2019). The morphology of reactive astrocytes gradually becomes more elongated, and their population density and GFAP expression gradually increase, reflecting their active role in the response to injury (Wanner et al., 2013). At 3 dpi, Nog-GFP signal increased in the peri-lesional region concomitant with rising GFAP immunoreactivity, resulting in partial co-localization within reactive astroglial areas (Figures 2E–H). By 7 dpi, both signals were prominently enriched along the lesion border, where numerous astrocytic profiles exhibited clear Nog-GFP/GFAP co-localization (Figures 2I–L). At 14 dpi, dense GFAP-positive processes delineated the lesion margin, consistent with a mature astroglial scar, and strong Nog-GFP signal persisted within this scar-associated region with frequent Nog-GFP/GFAP double-positive profiles (Figures 2M–P). Near the lesion site, the GFP-labeled Noggin-positive and GFAP-positive areas increased (Figures 2Q,R). Furthermore, we examined Noggin expression in microglia, which also actively respond to injury (Bradbury and Burnside, 2019). To exclude microglia as a potential cellular source of Noggin, we performed double immunofluorescence staining for GFP and Iba1 in sham-operated spinal cords and at 7 days post-injury. No apparent co-localization between Nog-GFP and Iba1 was observed at either time point (Supplementary Figure 2). These findings indicate that Noggin is predominantly expressed in GFAP-positive astrocytes within the glial scar after SCI. Notably, microglia are located not only at the glial scar after SCI, but also tend to accumulate within the lesion site (Hackett and Lee, 2016). These observations suggest that SCI induces Noggin expression during the early phase after injury and that this activity becomes enriched in GFAP-positive reactive astrocytes as the glial scar matures.

### 3.3 Induction of GFAP after SCI is attenuated in noggin cKO mice

**Figure 3:**
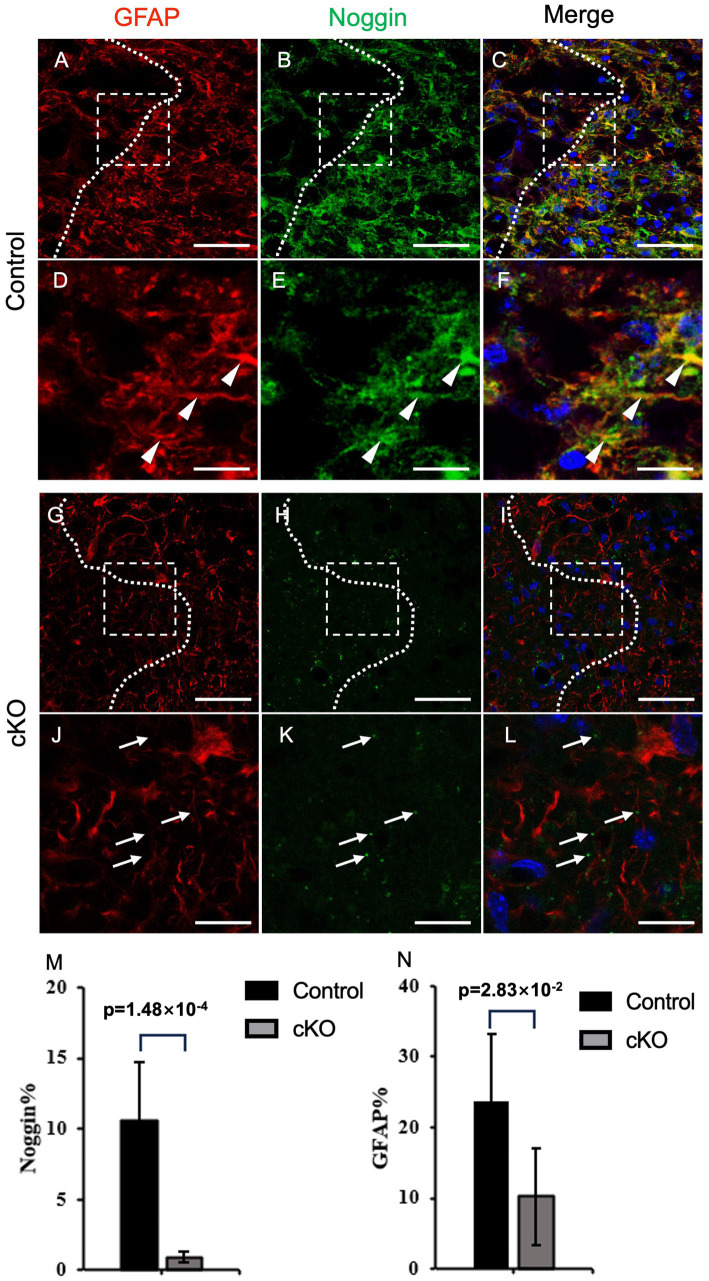
Inhibition of GFAP expression in *Nestin-Cre; Noggin^flox/flox^* cKO mice after spinal cord injury. **(A–F)** Immunohistological analysis of GFAP and Noggin in the control group after spinal cord injury. Noggin was co-expressed in GFAP positive astrocytes (arrowheads). **(G–L)** In cKO mice, Noggin and GFAP expression were weak and not colocalized (arrows). **(M)** GFAP expression was significantly lower in cKO mice. **(N)** Noggin expression was almost diminished in cKO mice compared (3 mice per group). Data are presented as mean ± SE. Dotted lines mark the injury boundaries. Dotted boxes mark the regions of interest for high-magnification display. Scale bars: 200 μm **(A–C,G–I)** and 50 μm **(D–F,J–L)**.

To clarify the role of endogenous Noggin in glial scars, we used *Noggin^flox/flox^; Nestin-Cre* cKO mice for the SCI model. After injury, reactive astrocyte proliferation occurs but ceases after approximately 2 weeks, when the glial scar matures (Yang et al., 2020). On day 14 post-SCI, we analyzed GFAP and Noggin expression (Figure 3). Noggin expression was upregulated outside of the core region in control mice (Figures 3A–F). Conversely, Noggin expression was significantly diminished in Noggin cKO mice (Figures 3H,K,M). However, Noggin expression was not completely abolished, indicating that a small amount of Noggin is probably secreted by non-neuronal/astrocytic cells. Indeed, the remaining signal did not colocalize with GFAP (Figures 3G–L). Interestingly, GFAP immunoreactivity was reduced in the cKO mice, suggesting that the astroglial response is attenuated in the absence of Noggin (Figure 3N). These results indicate that inhibition of BMP signaling is required for the complete induction of reactive astrogliosis following SCI.

### 3.4 Blockade of noggin inhibited GFAP expression and altered BMP signaling

**Figure 4:**
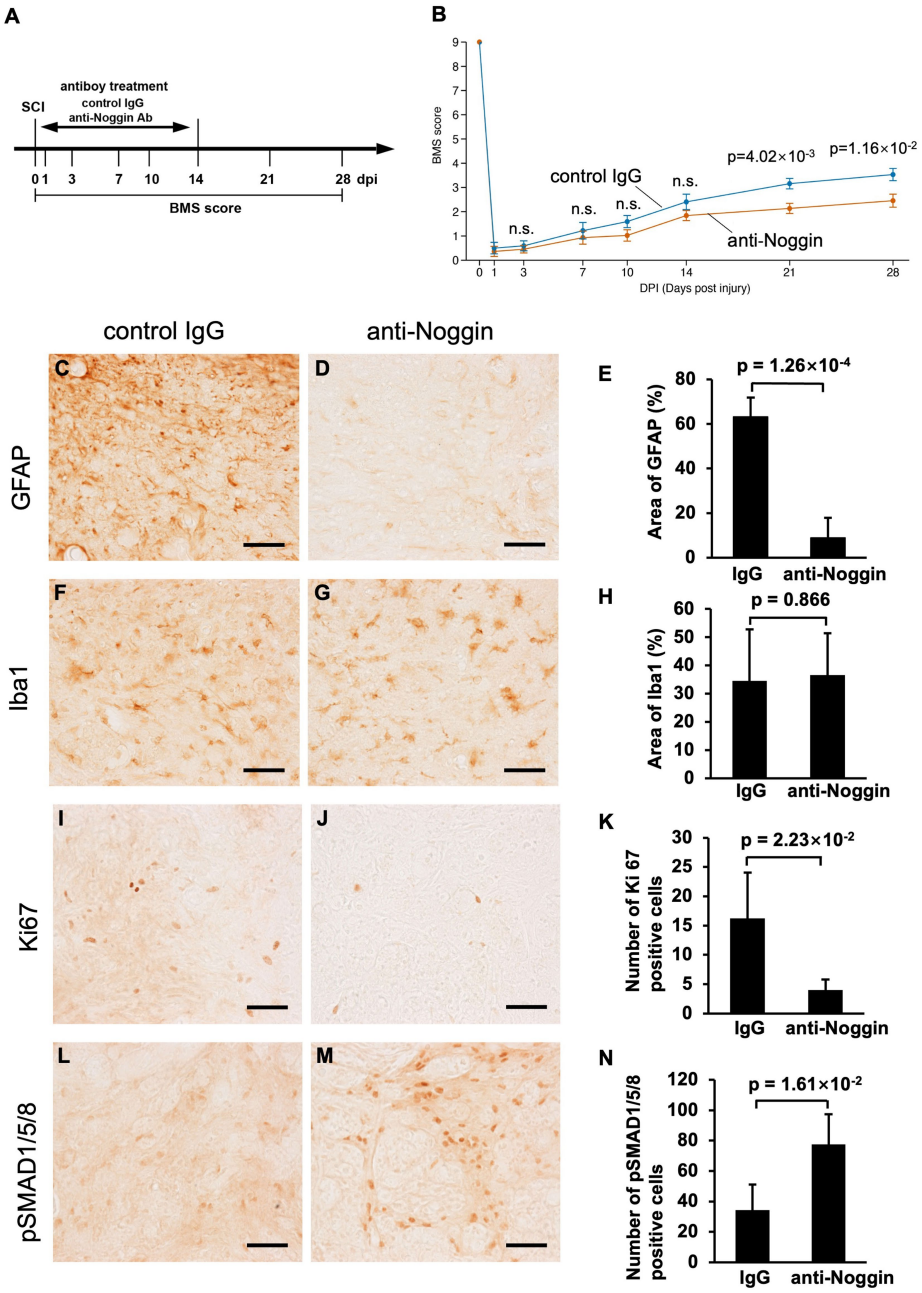
Anti-Noggin antibodies decrease GFAP expression after spinal cord injury. **(A)** Schematic of spinal cord injury (SCI) treated with antibodies. Control IgG- or anti-Noggin antibodies were administrated for 14 days. **(B)** Comparison of BMS scores in mice with SCI after treatment with control IgG or anti-Noggin antibodies. Indicate the number of mice (control IgG: 8 mice; anti-Noggin IgG: 11 mice). **(C–E)** GFAP expression in the control IgG- or anti-Noggin antibody-treated group 14 days after spinal cord injury. **(F–H)** Iba1 expression in the control IgG- or anti-Noggin antibody-treated group 14 days after SCI. **(I–K)** Ki67 expression in the control IgG- or anti-Noggin antibody-treated group 14 days after spinal cord injury. **(L–N)** pSMAD1/5/8 expression in the control IgG- or anti-Noggin antibody-treated group 14 days after SCI (4 mice per group). Data are presented as mean ± SE. Scale bars: 50 μm.

As the locomotor activity of cKO mice was highly affected because of abnormal bone formation in the lumbar region (data not shown), we could not perform behavioral tests before and after SCI. To perform loss-of-function analysis of Noggin in the SCI model, we administered the anti-Noggin antibody using an osmotic pump to neutralize Noggin for 14 days (Figure 4A). BMS scores showed that, compared to the IgG-treated control group, anti-Noggin antibody-treated SCI mice exhibited worse behavioral scores starting at 21 days and continuing until at least 28 days after injury (Figure 4B). Histologically, dense clusters of GFAP-positive cells were observed around the lesions, while the GFAP-positive signal was significantly reduced in the anti-Noggin antibody treatment group, which is consistent with the observation of the cKO mice (Figures 4C–E). There was no significant difference in the number of Iba1-positive cells between the control and anti-Noggin antibody treatment groups (Figures 4F–H). We also examined NG2 immunoreactivity as a representative CSPG-related marker at 14 dpi. NG2 signal was increased in the injured spinal cord of both groups and appeared more evident in the lesion core of the anti-Noggin antibody-treated group (Supplementary Figure 4). We next examined cell proliferation and BMP signaling activity by immunohistochemical staining for Ki67 and phosphorylated SMAD1/5/8 (pSMAD1/5/8). In the control group, a substantial number of Ki67-positive cells were detected around the lesion area at 14 days post-injury. In contrast, anti-Noggin antibody treatment resulted in a marked reduction in the number of Ki67-positive cells, suggesting decreased proliferative activity of astrocytes following Noggin neutralization (Figures 4I–K). While pSMAD1/5/8 immunoreactivity was relatively low in the control group, anti-Noggin antibody-treated spinal cords exhibited a pronounced increase in pSMAD1/5/8-positive cells within the injured region, indicating enhanced BMP–SMAD signaling activity upon Noggin blockade (Figures 4L–N).

## 4 Discussion

**Figure 5:**
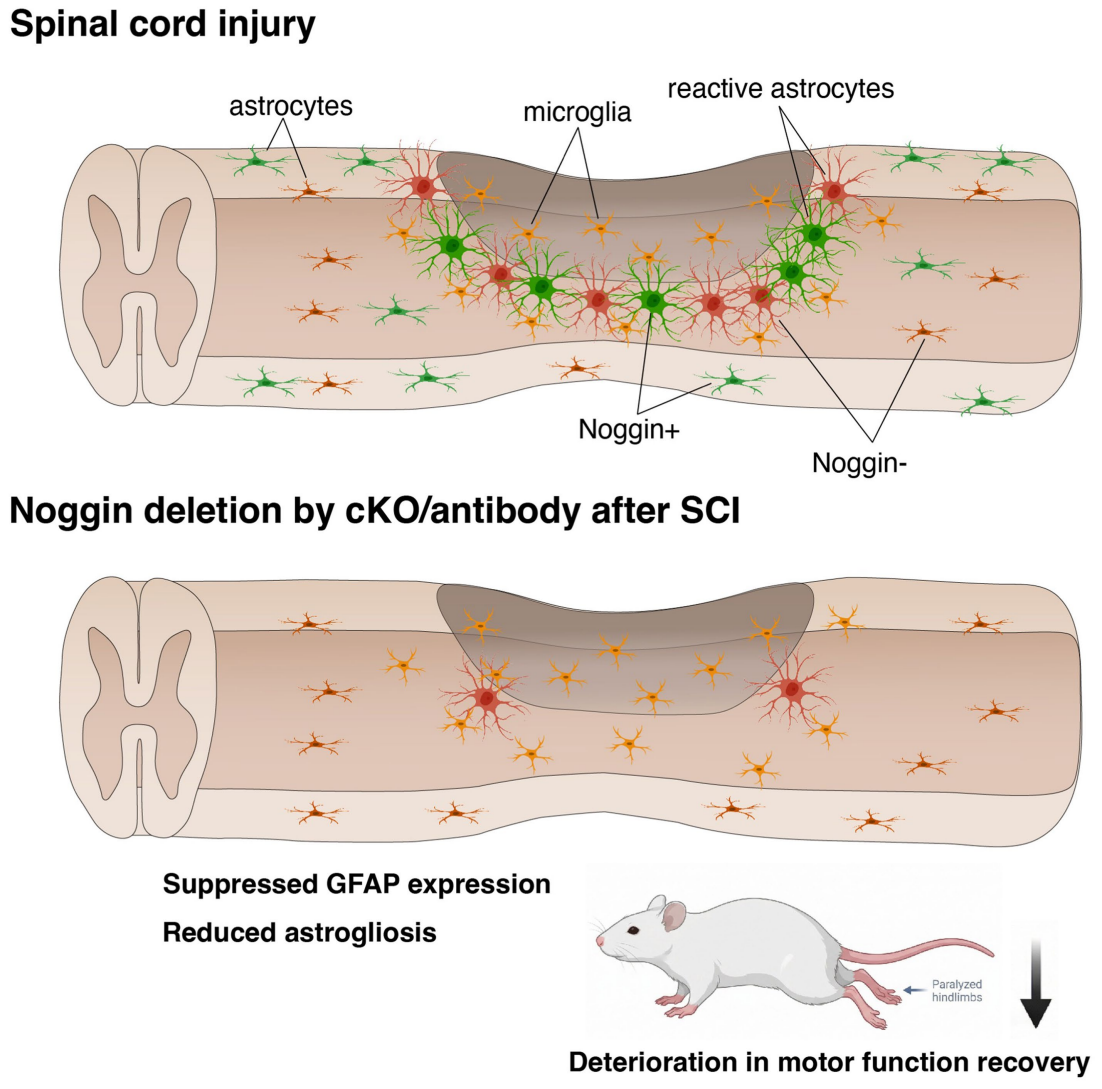
Schematic diagram of Noggin cKO and anti-Noggin antibody treatment after SCI. After SCI, Noggin expression is upregulated predominantly in GFAP-positive reactive astrocytes at the lesion border, where these cells contribute to glial scar formation. In contrast, deletion or neutralization of Noggin by cKO mice or anti-Noggin antibody treatment suppresses GFAP expression and attenuates astrogliosis after SCI. This reduced astroglial response is accompanied by deterioration of motor function recovery, suggesting that endogenous Noggin promotes reactive astrogliosis and contributes to tissue repair and functional recovery after SCI.

In this study, using Nog-GFP mice, we showed Noggin upregulation in astrocytes after SCI, consistent with a previous report (Hampton et al., 2007a). We showed that Noggin was mostly co-expressed with GFAP-positive astrocytes both before and after SCI. Loss-of-function using cKO mice and antibody neutralization showed inhibition of GFAP expression by depleting Noggin after SCI. In addition, we found that neutralizing Noggin suppressed functional recovery after SCI. Noggin expression disappeared from the injury site after SCI and gradually increased over time at the injury border, also known as the glial scar area, similar to previous reports (Wanner et al., 2013) (Figure 5).

It is known that BMPs are upregulated after SCI, which leads to activation of SMAD signaling pathways, inhibition of axonal growth and regeneration, and thus increased glial scar formation (Al-Sammarraie et al., 2023). Noggin acts as a BMP antagonist, binding to BMP and blocking its interaction with BMP receptors, inhibiting BMP-mediated cellular responses, and thereby promoting neuronal survival and growth, reducing scar tissue formation, and improving functional recovery after SCI (Enzmann et al., 2005; Xiao et al., 2010). It is also known that exogenous Noggin application prevents the differentiation of neural precursor cells into astrocytes by inhibiting BMPs and promotes the functional recovery of paraplegic mice (Setoguchi et al., 2004). The inhibitory effect of Noggin on astrocyte genesis is also classically reported in the case of neurogenesis in the hippocampal dentate gyrus and subventricular zone (Lim et al., 2000).

However, our findings suggest a more complex role for Noggin in regulating glial responses after spinal cord injury. Although Noggin is classically defined as an antagonist of BMP signaling, Noggin neutralization reduced GFAP immunoreactivity and Ki67-positive cells but was accompanied by enhanced pSMAD1/5/8 signaling. Thus, attenuation of GFAP-positive astroglial responses did not necessarily indicate improved tissue repair. Rather, anti-Noggin antibody treatment resulted in significantly worse functional recovery, as evidenced by reduced BMS scores in the anti-Noggin antibody–treated group. This difference became especially pronounced at 21 days post-injury, a stage corresponding to scar maturation and chronic tissue remodeling rather than the acute inflammatory phase (Bradbury and Burnside, 2019). These data indicate that endogenous Noggin may contribute to maintaining an appropriate BMP-signaling balance and preserving astrocyte-mediated protective responses during scar maturation after SCI. Consistent with this interpretation, BMP–SMAD activity has been reported to exert context-dependent effects on gliosis and tissue remodeling following SCI. In particular, distinct BMP receptor–mediated pathways can differentially regulate astrocytic reactivity and scar formation (Sahni et al., 2010). Therefore, Noggin may not simply act to suppress glial scar formation; instead, it may participate in balancing astroglial scar formation, BMP–SMAD activity, and functional stabilization after SCI.

Although anti-Noggin antibody treatment led to a reduction in GFAP immunoreactivity within the lesion border, this apparent attenuation of astrocytic reactivity did not translate into functional improvement. This observation is consistent with previous work demonstrating that inhibition of BMP signaling can enhance axonal growth and locomotor recovery without necessarily suppressing glial scar formation (Matsuura et al., 2008; Xiao et al., 2010). Notably, Matsuura et al. also showed that BMP inhibition by Noggin promotes axonal regeneration and functional recovery after SCI, while astroglial scar formation was not proportionally reduced, underscoring a dissociation between behavioral recovery and astrocytic scar markers (Matsuura et al., 2008).

A potential explanation for this dissociation lies in the multicellular nature of the spinal cord injury scar. Current models emphasize that the mature lesion comprises an astrocytic border surrounding a fibrotic and inflammatory core, with microglia playing a central role in coordinating inflammation, extracellular matrix remodeling, and intercellular signaling (Bradbury and Burnside, 2019; Tran et al., 2022). In this context, modulation of astrocytic GFAP expression alone may be insufficient to alter the overall trajectory of scar maturation and repair.

Consistent with this framework, we observed that while GFAP expression was reduced following Noggin neutralization, the number of Iba1-positive microglia was not significantly altered. This suggests that anti-Noggin antibody treatment preferentially affects astrocytic responses while leaving the microglial compartment numerically intact. Given the established bidirectional crosstalk between microglia and astrocytes during SCI, preserved microglial signaling may sustain a pro-inflammatory or pro-fibrotic microenvironment that counteracts the functional benefits of reduced astrocytic reactivity (Yuan and He, 2013; Tran et al., 2022).

Mechanistically, the combined reduction in Ki67-positive cells and increase in pSMAD1/5/8 immunoreactivity following anti-Noggin antibody treatment indicate enhanced canonical BMP signaling coupled with attenuated proliferative activity. BMP–SMAD signaling has been implicated in promoting astrocyte differentiation and cell-cycle exit rather than proliferation in the injured central nervous system (Sahni et al., 2010; Xiao et al., 2010). Notably, accumulating evidence indicates that astrocytes can actively support axonal growth and tissue repair after spinal cord injury (Anderson et al., 2016). Moreover, transplantation of pro-regenerative astrocytes into the injured spinal cord has been shown to promote axonal regeneration (Chang et al., 2023). In this context, Noggin neutralization may shift astrocytes toward a less proliferative state, as indicated by reduced Ki67 and enhanced BMP–SMAD signaling, resulting in decreased GFAP expression. Such a reduction in astrocytic proliferative capacity and/or astrocyte abundance may compromise the ability of astrocytes to provide sufficient structural and trophic support required for coordinated lesion stabilization and axonal protection. However, our study has several limitations. First, we were unable to directly quantify axonogenesis. Second, cKO mice have abnormal bone morphology of vertebrae at the lumbar and coccygeal regions, and thus we could not obtain their BMS score. There were no obvious differences in GFAP expression in the uninjured spinal cord of *Nestin-Cre; Noggin^flox/flox^* mice (Supplementary Figure 3).

CSPGs, including NG2, are important components of the post-injury extracellular matrix and have been widely implicated in glial scar formation and growth inhibition after SCI (McKeon et al., 1999; Bradbury et al., 2002; Jones et al., 2002; Tang et al., 2003). Because infiltrating bone marrow-derived macrophages have been reported as one possible source of NG2/CSPG4 after SCI (Liu et al., 2021), increased NG2 signal may partly reflect greater accumulation of NG2-expressing macrophage-lineage cells in the lesion core.

After injury, Noggin is mainly expressed in reactive astrocytes within the lesion area (Hampton et al., 2007b). As a BMP inhibitor, Noggin exhibits a higher affinity for BMP2/4 and a lower affinity for BMP7 (Lb et al., 1996; Balemans and Van Hul, 2002; Groppe et al., 2002). This may have inflammatory implications, given that BMP signalling has been reported to influence inflammatory responses in various pathological contexts (Nguyen et al., 2017; Elmadbouh and Singla, 2021). Following a central nervous system injury, reactive astrocytes play a role in both scar formation and the protection of remaining tissue. They maintain tissue structure, restrict the spread of inflammation and preserve neurological function (Faulkner et al., 2004; Linnerbauer and Rothhammer, 2020). Our results showed that pSmad1/5/8 signaling was enhanced in the anti-Noggin treatment group, while scar border-associated astrocytes in the lesion area were reduced (Figures 4C–E,L). In addition, the overall proliferative activity in the lesion area was decreased in the anti-Noggin treatment group (Figures 4I–K). One possible explanation is that Noggin blockade may disrupt the balance of BMP signaling after injury. Anti-Noggin treatment enhanced the activation of the canonical BMP-Smad1/5/8 pathway. In addition, Noggin-positive astrocytes play a role in the inflammatory response after injury. Inhibiting of Noggin may indirectly affects the ability of reactive astrocytes to restrict the spread of inflammation, which could impact inflammatory regulation and repair within the lesion microenvironment. Ultimately this may contribute to poorer neurological functional recovery in the anti-Noggin treatment group. Iba1- and CD206-positive cells were observed at the lesion border, and part of these cells appeared to accumulate around blood vessels (Supplementary Figure 5). However, the link between enhanced canonical BMP signaling and inflammatory imbalance remains mainly based on mechanistic inference and requires further experimental validation.

Another limitation of the present study is that the fidelity of GFP as a reporter for endogenous Noggin protein level was not comprehensively validated across all tissues and developmental stages. Immunostaining of Noggin in developing Nog-GFP mouse brain showed co-localization of GFP and endogenous Noggin in the cortex and hippocampus at P5, supporting the spatial consistency between reporter and endogenous protein in the developing brain (Supplementary Figure 6). However, this validation does not exclude the possibilitythat reporter activity may vary depending on tissue context or developmental stage. Although GFP signal may not fully reflect the absolute protein levels or extracellular distribution of endogenous Noggin in all tissues and developmental stages, it is still useful to monitor the promoter activity of Noggin.

In recent years, there has been increasing interest in developing strategies that modulate astrocytic responses after SCI to enhance neuronal regeneration and functional recovery. One approach is to promote the proliferation and transformation of responsive astrocytes, thereby promoting neuronal growth and regeneration. This can be achieved by activating specific signaling pathways, such as the STAT3 pathway (which regulates GFAP expression) or by using small molecules or other proteins that can regulate astrocytic gene expression and function (Okada et al., 2006). Noggin is not the only factor that regulates glial scar formation and astrocytic proliferation and differentiation. Therefore, further studies are needed to fully understand the complex molecular mechanisms underlying glial scar formation and its role in SCI recovery. Such knowledge could contribute to the development of therapeutic strategies for SCI and other neurological disorders.

## Data Availability

The datasets presented in this article are not readily available because they are available by contacting the corresponding author directly. Requests to access the datasets should be directed to SY, yamagish@hama-med.ac.jp.
