# Supplementary material for: Loss of function of Noggin inhibits glial scar formation and motor function recovery after spinal cord injury
Source: Front Neural Circuits. 2026 May 28;20:1821905. doi: 10.3389/fncir.2026.1821905 (PMC13253513; doi:10.3389/fncir.2026.1821905)
Supplement: Supplementary file 1 [file Data_Sheet_1.PDF]

Supplementary Figure 1

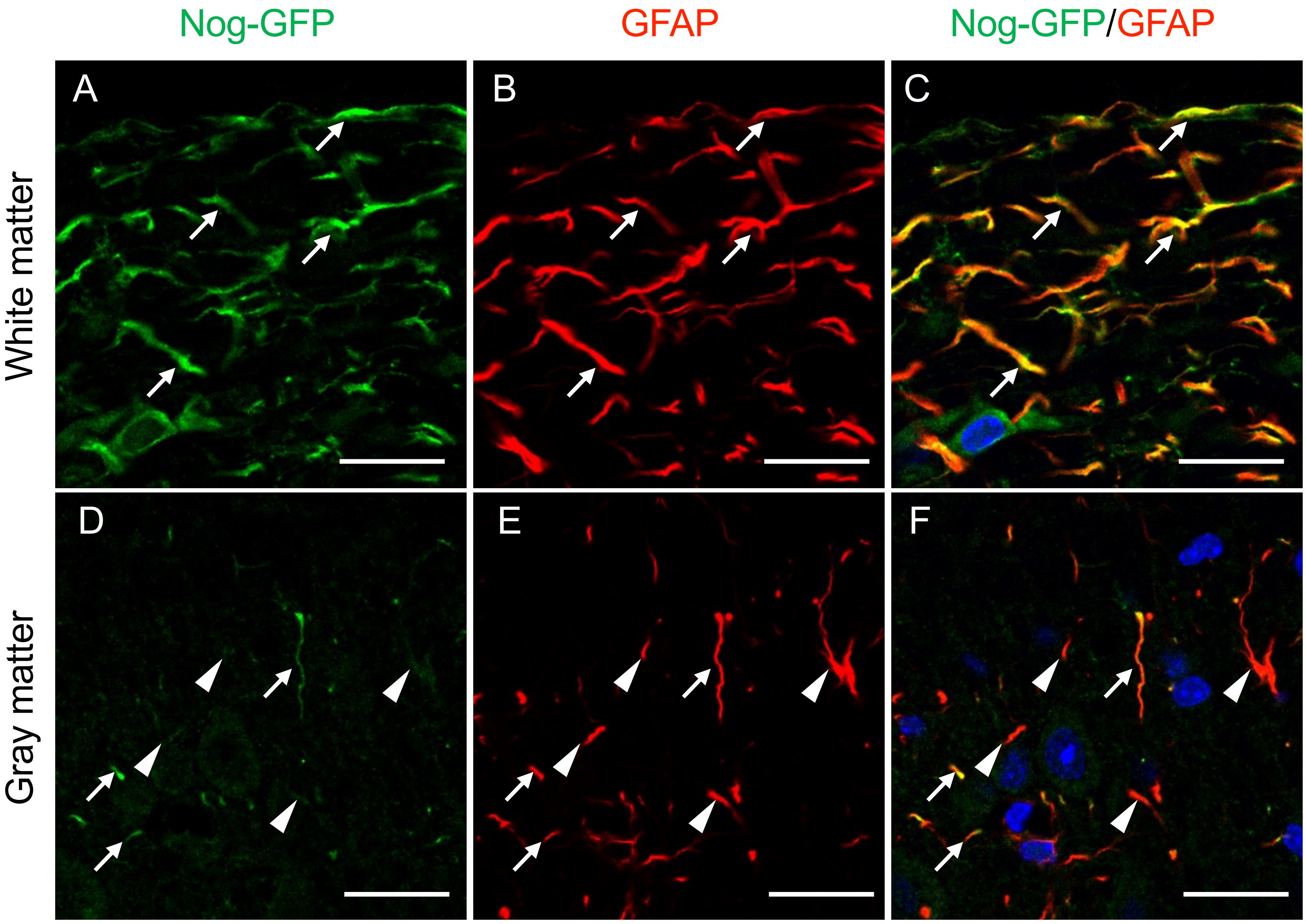

Supplementary Figure 1. Expression of Noggin in the spinal cord astrocytes. Representative immunohistochemical images showing GFP (green), GFAP (red), and DAPI (blue) in the white (upper) and gray matter (lower) of the spinal cord of Nog-GFP transgenic mice under sham conditions. Arrows: GFAP and Nog-GFP double positive cells. Arrowheads: GFAP single-positive cells. Scale bars: 50 µm.

Supplementary Figure 2

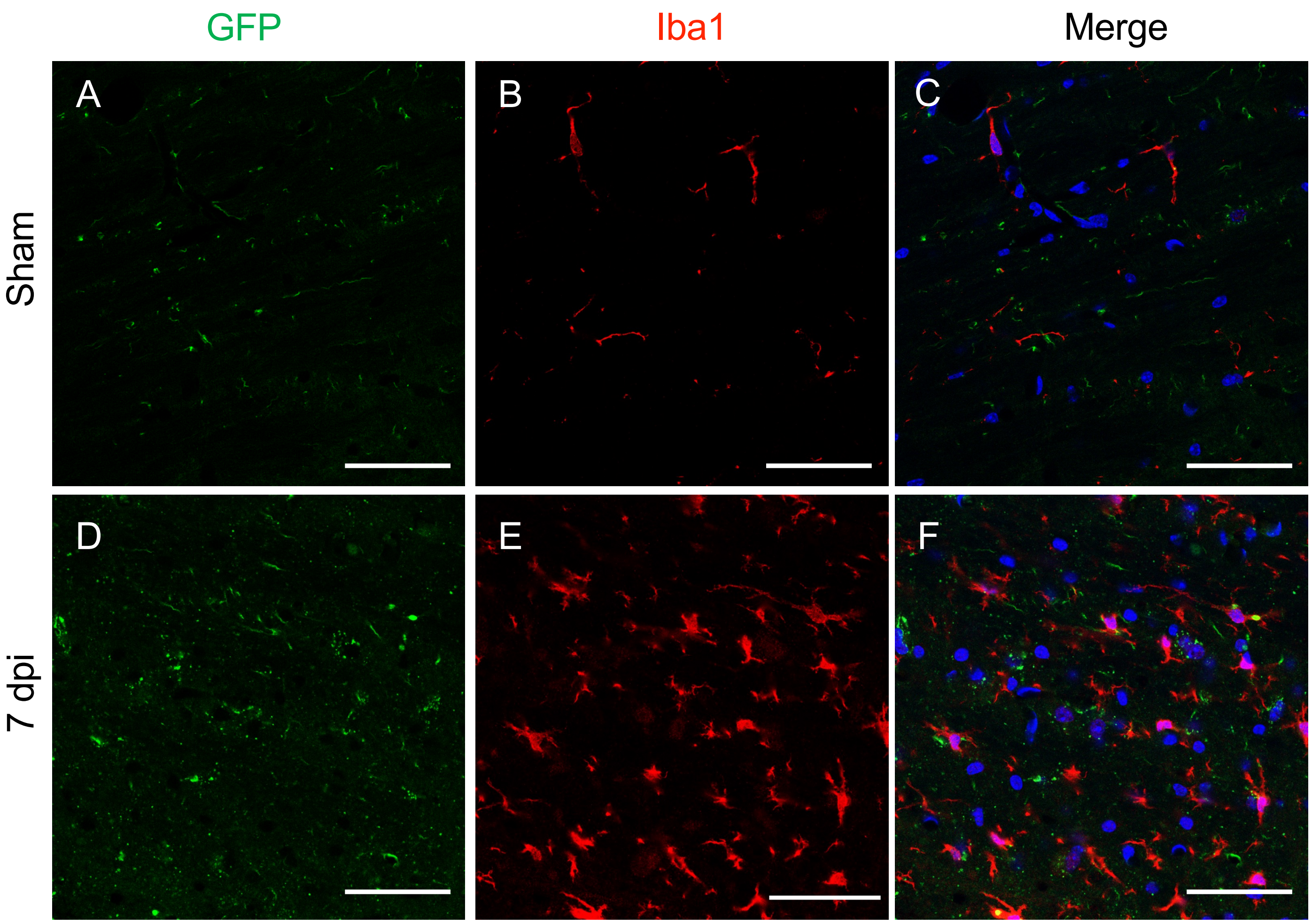

Supplementary Figure 2. Noggin is not expressed in microglia in sham and injured spinal cords.

Representative immunofluorescence images showing Nog-GFP (green) and Iba1 (red) staining in the spinal cord under sham conditions (A-C) and at seven days post injury (D-F). Please note that Nog-GFP and Iba1 signals do not overlap. Scale bars: 50 µm.

Supplementary Figure 3

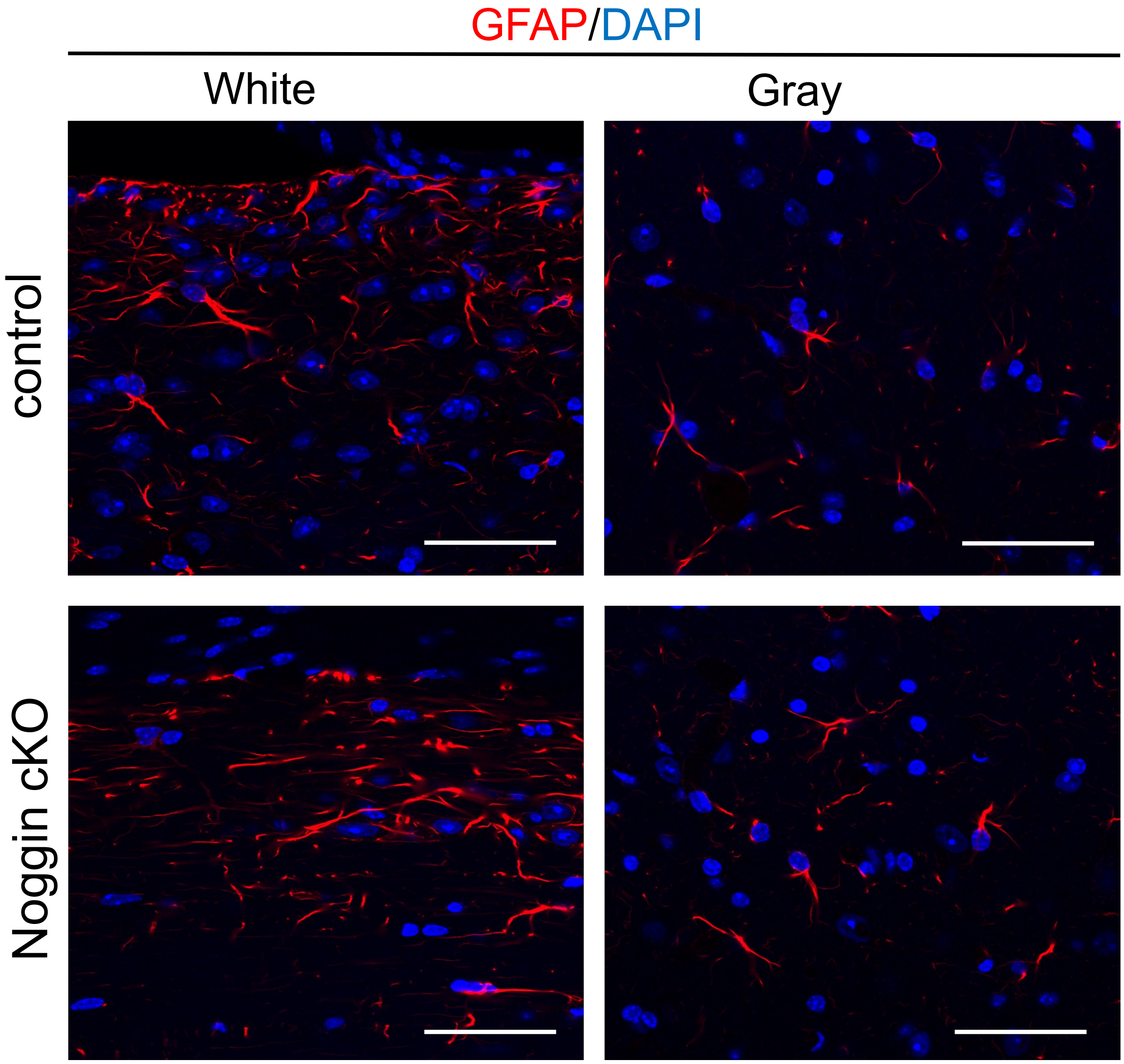

Supplementary Figure 3. Astrocytes are not altered in the spinal cord of uninjured Noggin cKO mice.

Representative immunofluorescence images showing GFAP (red) staining in the white and gray matter of the spinal cord under sham conditions in control (upper) and Noggin cKO mice (lower). Please note that no obvious abnormalities in astrocyte distribution or morphology were observed in cKO mice. Scale bars: 50 µm.

Supplementary Figure 4

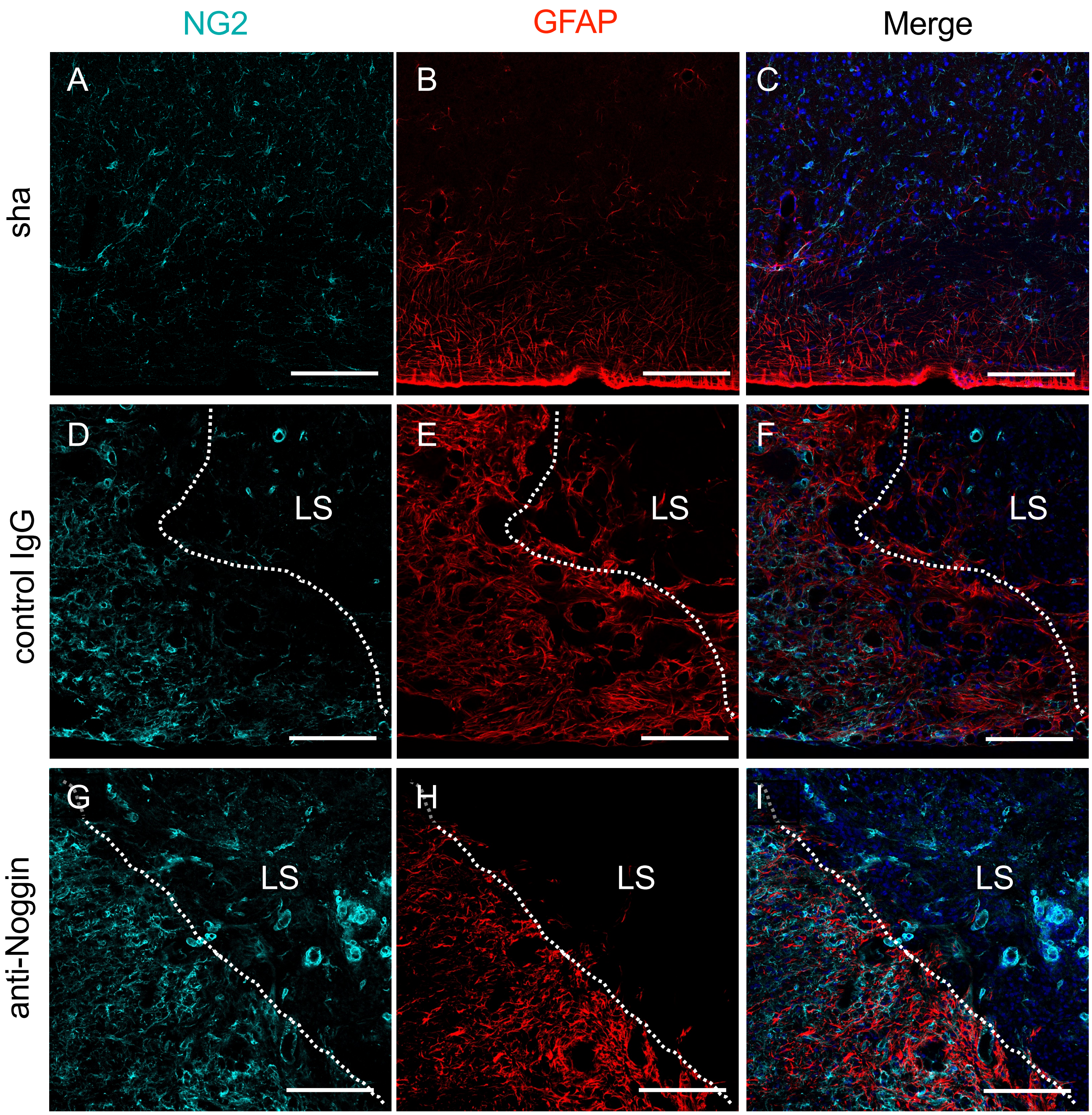

Supplementary Figure 4. NG2 immunoreactivity is increased in the lesion core of anti-Noggin antibody-treated spinal cords.

Representative immunofluorescence images showing NG2 (cyan) and GFAP (red) staining in the spinal cord under sham conditions, anti-IgG treatment and anti-Noggin IgG treatment at 14 days post-spinal cord injury. Dotted lines mark the injury boundaries. Please note that NG2 immunoreactivity was upregulated in both injured groups at 14 dpi and more prominent in the core region of the anti-Noggin antibody treated group. LS, lesion site. Scale bars: 200 µm.

Supplementary Figure 5

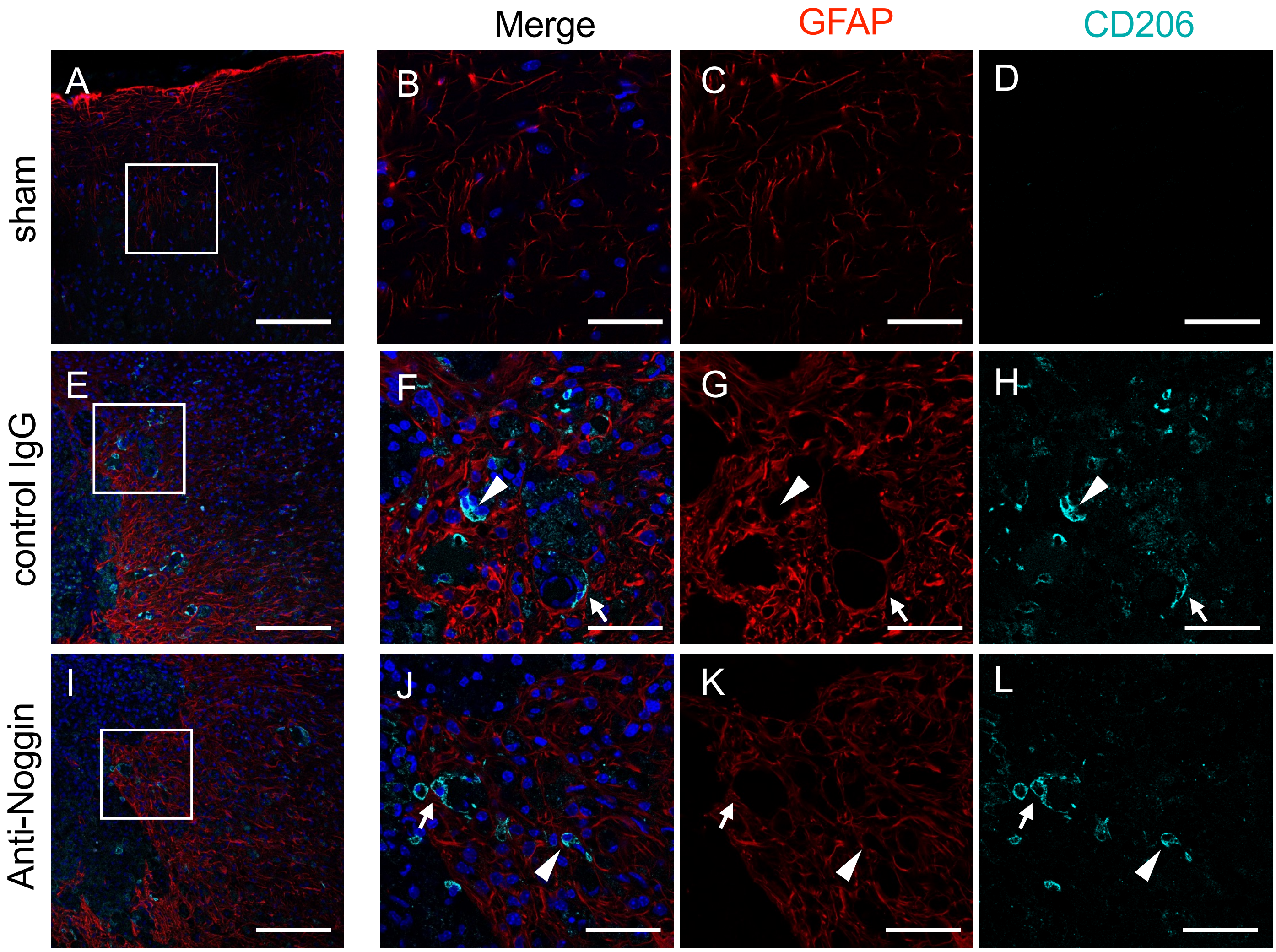

Supplementary Figure 5. CD206 expression in the spinal cord peri-lesional glial scar region. Representative immunofluorescence images showing CD206 (cyan) and GFAP (red) staining in the spinal cord under sham conditions (A-D), control-IgG treatment (E-H) and anti-Noggin IgG treatment (I-L) at 14 days post-spinal cord injury. Arrows indicate the CD206-positive signal around blood vessels. Arrowheads indicate CD206 positive signal in the non-vascular regions. Scale bars: 200 µm (A, E, I) and 50 µm (B–D, F–H, J–L).

Supplementary Figure 6

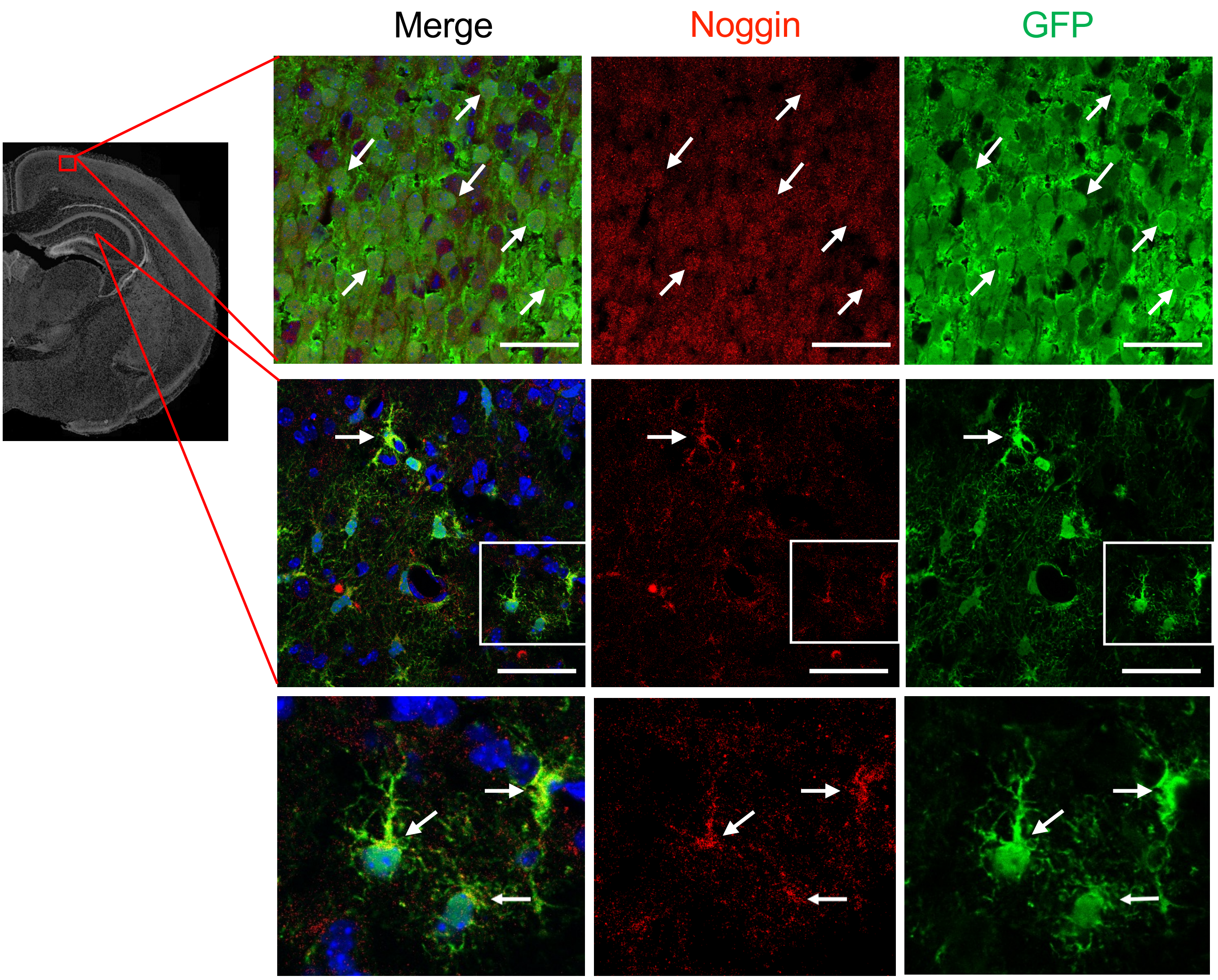

Supplementary Figure 6. GFP signals represent Noggin protein expression in the developing mouse brain.

Representative immunofluorescence images showing Nog-GFP (green) and Noggin immunostaining (red) in the cerebral cortex (upper) and the hippocampus (middle and lower) at P5. Please note that the expression patterns of Nog-GFP and endogenous Noggin were similar. Arrows: GFP and endogenous Noggin positive cells. Scale bars: 50 µm.

Supplementary Movie 1

Representative 3D image of GFP (Noggin; green) and GFAP (red) immunostaining in the uninjured spinal cord. Noggin is strongly expressed in the white matter, most of which overlaps with GFAP.
